# Language-model-based patient embedding using electronic health records facilitates phenotyping, disease forecasting, and progression analysis

**DOI:** 10.21203/rs.3.rs-4708839/v1

**Published:** 2024-09-23

**Authors:** Su Xian, Monika E. Grabowska, Iftikhar J. Kullo, Yuan Luo, Jordan W. Smoller, Wei-Qi Wei, Gail Jarvik, Sean Mooney, David Crosslin

**Affiliations:** 1 Department of Biomedical Informatics and Medical Education, University of Washington, Seattle, WA; 2 Department of Medicine, Division of Medical Genetics, University of Washington, Seattle, WA; 3 Department of Genome Sciences, University of Washington, Seattle, WA; 4 Department of Medicine, Division of Biomedical Informatics and Genomics, Tulane University, New Orleans, LA; 5 Department of Biomedical Informatics, Vanderbilt University Medical Center, Nashville, TN; 6 Psychiatric and Neurodevelopmental Genetics Unit, Center for Genomic Medicine, Massachusetts General Hospital, Boston, MA; 7 Center for Precision Psychiatry, Department of Psychiatry, Massachusetts General Hospital, Boston, MA; 8 Department of Cardiovascular Medicine and the Gonda Vascular Center, Mayo Clinic Rochester Minnesota; 9 Department of Preventive Medicine, Northwestern University Feinberg School of Medicine; 10 Center for Information Technology, National Institutes of Health

## Abstract

Current studies regarding the secondary use of electronic health records (EHR) predominantly rely on domain expertise and existing medical knowledge. Though significant efforts have been devoted to investigating the application of machine learning algorithms in the EHR, efficient and powerful representation of patients is needed to unleash the potential of discovering new medical patterns underlying the EHR. Here, we present an unsupervised method for embedding high-dimensional EHR data at the patient level, aimed at characterizing patient heterogeneity in complex diseases and identifying new disease patterns associated with clinical outcome disparities. Inspired by the architecture of modern language models—specifically transformers with attention mechanisms, we use patient diagnosis and procedure codes as vocabularies and treat each patient as a sentence to perform the patient embedding. We applied this approach to 34,851 unique medical codes across 1,046,649 longitudinal patient events, including 102,739 patients from the electronic Medical Records and GEnomics (eMERGE) Network. The resulting patient vectors demonstrated excellent performance in predicting future disease events (median AUROC = 0.87 within one year) and bulk phenotyping (median AUROC = 0.84). We then illustrated the utility of these patient vectors in revealing heterogeneous comorbidity patterns, exemplified by disease subtypes in colorectal cancer and systemic lupus erythematosus, and capturing distinct longitudinal disease trajectories. External validation using EHR data from the University of Washington confirmed robust model performance, with median AUROCs of 0.83 and 0.84 for bulk phenotyping tasks and disease onset prediction, respectively. Importantly, the model reproduced the clustering results of disease subtypes identified in the eMERGE cohort and uncovered variations in overall mortality among these subtypes. Together, these results underscore the potential of representation learning in EHRs to enhance patient characterization and associated clinical outcomes, thereby advancing disease forecasting and facilitating personalized medicine.

## Introduction

Electronic health records (EHR) have been widely adopted in the United States^1^. The “meaningful use” of EHR, released by the Department of Health and Human Services, aims to improve the quality and efficiency of care^2^. To also facilitate clinical research, the potential of the secondary use of EHR has been explored and drawn large attention in recent decades. One of the major topics is EHR-based digital phenotyping^3–5^. The electronic MEdical Records and GEnomics (eMERGE) consortium launched Phenotype KnowledgeBase (PheKB) as a digital phenotyping knowledge base that engages multiple sites of large hospitals and universities to share their phenotyping algorithms developed using the EHR data^6,7^. Though most of the algorithms stored in PheKB are rule-based and validated by domain expertise, there are also efforts towards developing scalable machine learning algorithms for EHR-based phenotyping, which generally require less time and expertise. Various machine learning and deep learning methods have been applied to build EHR-based phenotyping algorithms, including support vector machines (SVM), random forests, logistic regressions, and neural network architectures^3,8,9^. These efforts are marching towards a better characterization of diseases and aim to build a better healthcare system. Currently, a crucial and challenging question is how to leverage the EHR data to help identify and characterize disease patterns. With the ideal solution, we can promote disease monitoring and clinical predictive tasks.

Representation learning is a powerful tool that can characterize existing and uncover novel disease patterns to facilitate the study of disease etiology, prevention, forecasting, and even heterogeneity analysis^10–13^/ Though researchers in modern times are trying to find new patterns across the EHR data for different diseases^14^, the secondary use of EHR research heavily relies on existing domain knowledge from expertise^15–18^. There are emerging new findings using the EHR to unfold heterogeneity within a defined phenotype, revealing differences in comorbidities potentially linked with genetics, lifestyle, and environmental factors^19–21^. To further improve this, representation learning has emerged as one crucial tool dealing with high dimensional EHR data to facilitate pattern recognition, heterogeneity analysis, and downstream prediction tasks. A big challenge is that, in the EHR, each patient can have multiple visits in a year or across different years, generating abundant diagnosis and procedure codes accompanied by numerous lab values and observations, making it difficult to summarize. A simple representation of patients utilizing the nature of the EHR data structure is through binary vector representation to indicate if patients have specific diagnoses, procedures, or labs. Under these circumstances, matrix decomposition becomes a convenient tool to encode patients into relatively lower numerical spaces^22^. Matrix decomposition, such as principal components analysis (PCA) and non-negative matrix factorization (NMF), is used to compress a large matrix of patients into a relatively smaller one, while preserving the relationship of the selected features, such as diagnosis, procedures, and labs. The output matrix is usually termed patient embedding. Besides traditional matrix decomposition, a specific deep-learning model adopted from the autoencoder has been implemented to perform the embedding task^10^. Apart from the purely data-driven method, some methods integrate medical entities to perform predictive tasks^23^. Overall, without the obligation of cohort building or domain expertise to iteratively process the phenotyping tasks, various unsupervised methods are applied to identify unknown patterns of patients and diseases using the EHR data.

Many neural network architectures have been developed and proven to show progress in various kinds of health-related tasks, both supervised and unsupervised^24,25^. Specifically, transformer attention mechanisms have surpassed the traditional sequence models, such as recurrent neural networks (RNN) and some of their variations (gated recurrent unit [GRU], long short-term memory [LSTM]), both in efficiency and performance in downstream tasks within the natural language processing (NLP) field^26,27^. Inspired by the attention mechanisms, we explored the potential of using medical diagnosis and procedures to construct patient vectors. In this work, we represented each patient as a sentence, using diagnosis and procedure codes as vocabularies to compose patient vectors. Moreover, we generated patient vectors in a longitudinal format, allowing investigation of a patient at a specific time point. Here, we present the language-model-based EHR patient data embedding method and demonstrate its power in bulk phenotyping, future disease prediction, comorbidity study, and longitudinal analysis. Using 34,851 available codes for 1,046,649 patients from the electronic MEdical Records and GEnomics (eMERGE) network, we first trained the model and generated the embeddings of patients. The model achieved outstanding performance in predicting future disease events (median ROC = 0.87) and bulk-phenotyping (median ROC = 0.84), using phecodes as a standard reference^28^. More importantly, we showed that patient vectors can reveal the heterogeneity of comorbidity patterns within a single phenotype, which forms sub-clusters of comorbidities. We demonstrated that these clusters have different disease progression trajectories longitudinally. With external validation using de-identified patients from UW across 20 years (n = 840,000, from 2000 to 2020), our model showed great flexibility and stable performances in reproducing the sub-cluster analysis and identifying distinct clinical outcomes associated with each cluster. Together, these results bring new insight into personalized medicine by analyzing complex comorbidity patterns within diseases and revealing unique disease trajectories linked with varied mortality.

## Materials and Methods

### Data

Patient EHR data from the eMERGE Network (n = 102,740) were used for training and building the patient embedding models. These included basic demographics (birth decade, gender, race, and ethnicity), patient diagnosis codes (ICD-10 and ICD-9), procedure codes (CPT-4), and age at diagnosis. The UW EHR data (n = 840,000) served as a validation set. Besides the same data elements mentioned above (birth decade, gender, race, ethnicity, diagnoses, and procedures), UW included overall mortality data, which is then used to evaluate the survival differences among clusters.

### IRB

This study is approved by the IRB STUDY00015886: Generalizability Assessment of the eMERGE study on the University of Washington Medical Center Population. All data extracted from the University of Washington is under this IRB approval and stored on the HIPAA-compliant server.

### Autoencoder model structure

**Figure 1.**
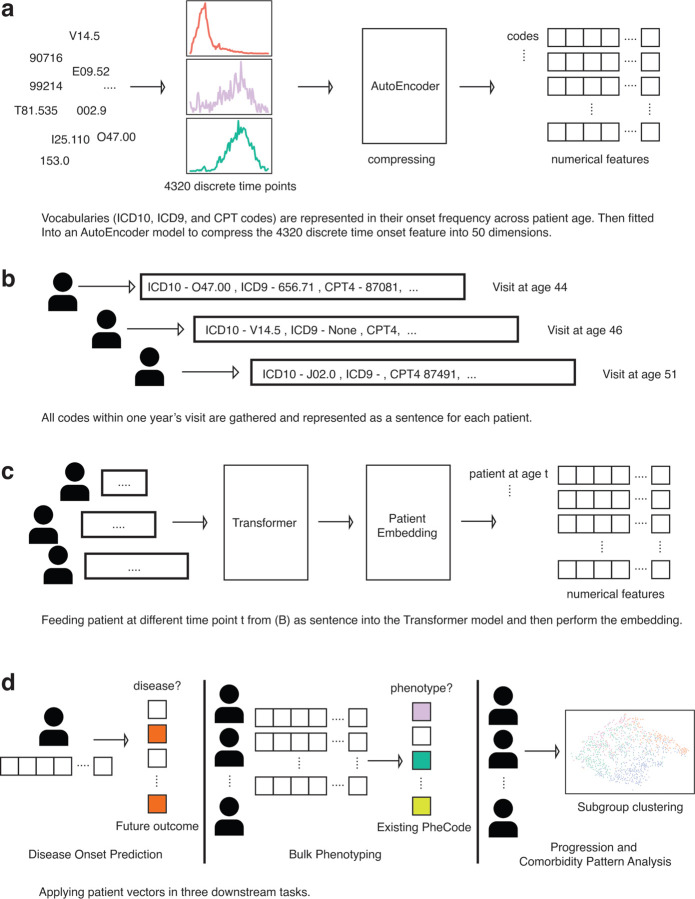
Illustration of the patient embedding model and downstream application a. Encoding diagnosis and procedure codes into numerical space as basic vocabularies in downstream training using an Autoencoder architecture. b. Representing patients’ visits within a year as sentences and diagnosis, procedure codes as vocabularies. c. Feed each visit from (b) into a Transformer model and concatenate through Patient Embedding model to generate the final patient vectors. d. Downstream applications workflow of disease onset prediction (left), bulk phenotyping (middle), and clustering subgroup analysis (right)

We collected all available codes, including ICD-9, ICD10, and procedure codes. To ensure the embedding quality, we kept all codes that appeared more than five times in the entire dataset, resulting in 34,851 unique codes. We used the week as a time interval unit and counted the number of appearances of each code across 0 to 4320 weeks, which equals 90 years. This produced a 34,851 × 4320-dimensional matrix, with each unique code represented by a vector of length 4320, and in the autoencoder model, this served as a feature for each code. The autoencoder model architecture is illustrated in Figure 1. The goal of this model is to condense the representation of the codes, producing a 34,851 × 50-dimensional matrix, where each code vector length is reduced from 4320 to 50. The non-linear functions in the autoencoder can identify complex patterns among the codes. Apart from using the traditional reconstruction error to train the model, we added the cosine similarities loss function to preserve some linear correlation properties among those codes. Moreover, the embedded layer contains a mean and standard deviation parameter, making this a variational autoencoder. We trained this model with Adam optimizer ^29^ with default parameters of beta1 and beta2, learning rate 1e-7, and batch size of 64 (Figure S1). During the training, 5% of the training data is split and used as a validation set to evaluate the loss. The model is trained for 100 epochs and reaches a steady reconstruction loss and similarity loss. The embedded 50-dimensional codes are then served as vocabularies in the transformer model to construct patient vectors.

### Transformer models structure

We adopt the transformer model, with the attention mechanism to build patient vectors ^26,27^. Our model uses a standard transformer model architecture (Figure S1), except that we did not include the positional embeddings for each code. Diagnoses often do not directly indicate the exact disease onset time. It only denotes the starting point of potential treatment and systemic awareness of the presence of diseases, as many chronic diseases indeed happen way earlier than a hospital diagnosis. Thus, all events documented in the hospital within a specific year do not suggest the absolute sequential relationship in real life. Therefore, we binned all codes for a patient happening within a year into a single vector, we do not meticulously require a specific positional encoding of codes within a particular year. The relative sequence within a specific year. We use the hyperparameter L = 6, H = 10, diff = 2048, and d = 200. The pre-trained embeddings of codes from the autoencoder serve as vocabularies. In the preprocessing step, we bin each patient’s codes by year, and for each year, we create a vector of codes to represent the temporal patient vector. The maximum vector length is set to be 250, considering that in one year, most patients should receive less than 250 codes from the hospital. For all 102,740 available patients, only 0.25% have more codes than 250 in a year, which is rare and might only happen to extremely severe patients. For patients having codes less than 250, we apply zero padding to the sequence and mark it as a special padding token. In total, there are 1,046,649 patient vectors for 102,740 patients. The goal of this transformer model is to use the current year’s codes to predict the future year’s codes, therefore, learning the relationship and progression longitudinally. Thus, we fit longitudinal patient events codes at year i as input, and the patient events at year i+1 as output. During the training process, we masked 20% of the codes in each vector and let the model reconstruct the full sequence of vectors. We trained this model with Adam optimizer with beta1 = 0.9, beta2 = 0.98, epsilon = 1e-9, and a scheduled learning rate gradually decreased to 0.002 at step 10,000. The batch size is set to 32, reducing the computation memory load. Again, 5% of the data is split and used as validation to evaluate the loss during the training.

### Sentence model structure and training detail

Inspired by the sentence embedding method ^30^, we build a similar model architecture that takes two patients as input and gives two binary outputs. The first binary output denotes whether the two vectors of patients belong to the same patient $\left(is\_same\_patient\;\text{task}\right)$. The second one evaluates if the one vector is the following event of another vector$\left(is\_next\_event\;\text{task}\right)$. We first use the global average pool of the embedding layer from the transformer model to represent patients as numerical vectors (Figure 1). We then added a feed-forward structure of a pre-embedding layer and a 50-dimensional embedding layer to compute the complex interactions of the embedded sequence (Figure 1). We use two different loss functions to optimize the two tasks mentioned above. The goal of the is_same_patienttask is to minimize the mean square distance between two 50-dimensional embedded vectors, as an evaluation of the vector distance in geometric space. While the is_next_eventtask is optimized by a 2-layer feed-forward neural network constructed by concatenating the embedding of two vectors, this might allow learning of complicated relationships among two vectors. We only used two layers of feed-forward structure to avoid overfitting and to ensure it learned meaningful functions according to the theory that two layers of neural networks can simulate any form of continuous function. During the training, we randomly formed pairs of patient vectors as input and trained until no improvement (loss decrease). We repeated the training process a few times, considering the variation due to the randomness of parameter initialization. We found the model performance peaked at 5000 steps during training with a learning rate = 3e-4.

### Mapping of diagnosis code to phecodes

The mapping between ICD9 and ICD10 diagnosis codes to phecodes is done by referencing the PheWAS website https://phewascatalog.org/ through the phecodes mapping panel, counting at least one presence of diagnosis code as qualifying phenotypes.

### Disease onset prediction and bulk phenotyping

Both disease onset prediction and bulk phenotyping are classification tasks in this work. In the disease onset prediction, for each phenotype, we collected all longitudinal vectors of patients and split them into before the onset versus after the onset group. We then used the longitudinal vectors to build a logistic regression classifier to perform classification tasks for the two groups of vectors. For example, for each diseasei, for j in 1…50, $x_{j}$represents numerical features drawn from the embedding (embedding size of 50), the logistic regression prediction whether the disease i is already presented in the longitudinal vector or not:

$$onset_{i}=\frac{1}{1+e^{\sum -\beta_{j}x_{j}}}$$

Bulk phenotyping is less complicated. For each phenotype $\left(phenotype_{i}\right)$, we computed the mean of vectors $\left(meanVector_{k}\right)$ across all time point $\left(t\right)$ within individual patients $\left(patient_{k}\right)$, resulting in a single vector for each patient

$$meanVector_{k}=\frac{\sum_{\left(t=1\ldots n\right)}patientVector_{k}^{t}}{n}$$

Logistic regression models were then applied to these vectors to discern whether patients exhibit a specific phenotype or not, as defined by phecodes.


$$phenotype_{i}=\frac{1}{1+e^{\sum -\beta_{j}x_{j}}}$$

### Comorbidity cluster analysis

We performed comorbidity analysis within a single phenotype to reflect the heterogeneity. Gaussian mixture model is used to first group samples into clusters. We chose the number of clusters based on the Bayesian information criteria (BIC). Then, within individual clusters, we performed logistic regression for each comorbidity (defined by phecodes), including the cluster (using one versus the rest), age of onset, sites, gender, ethnicity, and race as covariates to predict the comorbidity. e.g.


$$phecode_{i}=\frac{1}{1+e^{\beta_{0}+\beta_{1}age+\beta_{2}sex+\beta_{3}sites+\beta_{4}gender+\beta_{5}ethnicity+\beta_{6}cluster_{c}}}$$

Adjusting for multiple tests (n = 1,855 comorbidities), we used Benjamini-Hochberg adjusted p-values < 2e-5 as the significance level.

### Model evaluation

We evaluated the external performance of the model on both the eMERGE dataset (internal) and the UW EHR data (external). As described in the model architecture session, there are 1,046,649 patient vectors for 102,740 patients for the eMERGE dataset. Externally, we pulled available de-identified UW EHR data from the year 2000 to 2020, including n = 840,000 patients. We evaluated the transformer model and the patient-vector model output. For eMERGE data, we randomly selected 32,000 events (1000 steps with batch size 32) for the evaluation task. For UW data, the evaluation of the transformer model used randomly drawn 5000 patients, consisting of n = 66,776 events in total. The goal was to investigate the ability of the model to reconstruct the original codes given the patient vector. Performance for the eMERGE dataset is included in the Results Section and Table 1. Performance for the UW dataset is available in Table 3. The S-BERT model evaluation used 500 randomly drawn patients in 5 iterations (100 randomly drawn patients in each iteration), consisting of 34,633 and 29,587 events for eMERGE (Table 2) and UW datasets (Table 4), respectively. Note that the evaluation process is based on the number of events, not the number of patients. Thus, though the number of patients is relatively small compared to the total sample size, the number of events already reflected consistent and robust performance. Moreover, the summary statistics of the evaluation metrics demonstrated extremely low variance, suggesting robustness against large numbers of patients.

### Longitudinal comorbidity analysis

For each patient, the longitudinal vectors after the disease onset are collected. We used 10 years as the end point of disease progression, meaning that we collected 10 longitudinal vectors for each available patient (n = 110), using CRC as an example. We again computed the average of the vectors within individual patients and used them to perform the PCA (Figure 7b). We defined the occurrence of phenotypes as phenotypes that only occur after the onset of disease, which did not exist before the disease onset. The new phenotypes were first counted and then normalized by the number of patients within each cluster, represented as frequency. We then selected the top 15 phenotypes within each cluster and represented them in the plot (Figure 7c)

### Software versions and code availability

The patient embedding model is implemented through tensorflow version 2.3.0, with mostly the high-level API tensorflow.keras, version 2.4.0. Models were trained using an NVIDIA 2060-Super GPU with 8 GB RAM. The code for running the model and synthetic data are available at the GitHub repo https://github.com/suxian06/language-model-based-patient-embedding/tree/main. Data analysis using TSNE, PCA, GMM, and BIC was implemented through the scikit-learn version package 0.24.2 in Python. Logistic regression and analysis of variance (ANOVA) were implemented using statsmodels, version 0.12.2. Plots were generated using Matplotlib version 3.4.3, Seaborn 0.11.2. The online interactive charts were generated using Altair version 5.0.1. Survival analyses (Kaplan-Meier plot and Log-rank test) were performed using the scikit-survival packages in Python, version 0.14.0. Log-rank test used in differentiating the subgroup survival were also based on the scikit-survival package (compare_survival function). Standardization, numerical operations, and data cleaning are done with numpy version 1.23.0 and scipy version 1.6.2.

## Results

### Model illustration and performance evaluation

The outline of our work is summarized in Figure 1, with detailed model architecture in Figure S1. The three steps of performing numerical patient embedding in this session is illustrated in Figure 1a-c. We first created an embedding of vocabularies (diagnosis codes, procedure codes) using a variational autoencoder neural network architecture (Figure 1a, Figure S1). Then, we fed the embedded diagnosis codes and procedure codes as vocabularies to a transformer model, representing each patient’s longitudinal visits as sentences (Figure 1b, c, Figure S1). Lastly, we adopted the sentence-BERT architecture, flattening the 2-D vector output (vocabularies x probability matrix) from the transformer model into a 1-D vector as the final output of patient embedding (Figure 1c, Figure S1). We evaluated each step separately with more focus on the last two steps (Figure 1b, c), as they are influential for the performance of crucial downstream tasks.

During training, the transformer model masked 20% of the codes, and we evaluated the ability to use the existing codes and predict the future next year’s codes yet the model peaked at 79% prediction accuracy. The result suggests that the learned numerical embeddings can almost perfectly recapture what is shown in the training set. While inferring the 20% of masked code based on giving information is still challenging. During the evaluation, we randomly selected 32,000 samples (1000 steps with batch size 32) without masking the sequence, and the model achieved 91.7% mean precision and 95.8% median precision, with 89.5% mean recall and 93.7% median recall (Table 1) in reconstructing the original codes. These results demonstrate that the numerical embedded vector can accurately recover the diagnosis and procedure codes.

The sentence-embedding model (Figure S1) has two goals. One is to predict whether a given pair of patient embeddings at different time points represents the same patient. Another goal is to predict if one patient embedding is a longitudinal following event of another. During the evaluation, we selected 34,633 events from 500 randomly selected patients, forming pairs as input and dividing them into 5 iterations (100 patients for each iteration), and evaluated the performance of two tasks (Table 2). The accuracy for the is_same_patienttask is 0.797±0.0016 (mean and standard deviation), and for is_next_event is 0.769±0.011. Overall, the model achieved ideal performances on two crucial tasks, indicating a great potential for multipurpose downstream analysis.

### Disease onset time prediction and bulk-phenotyping

**Figure 2.**
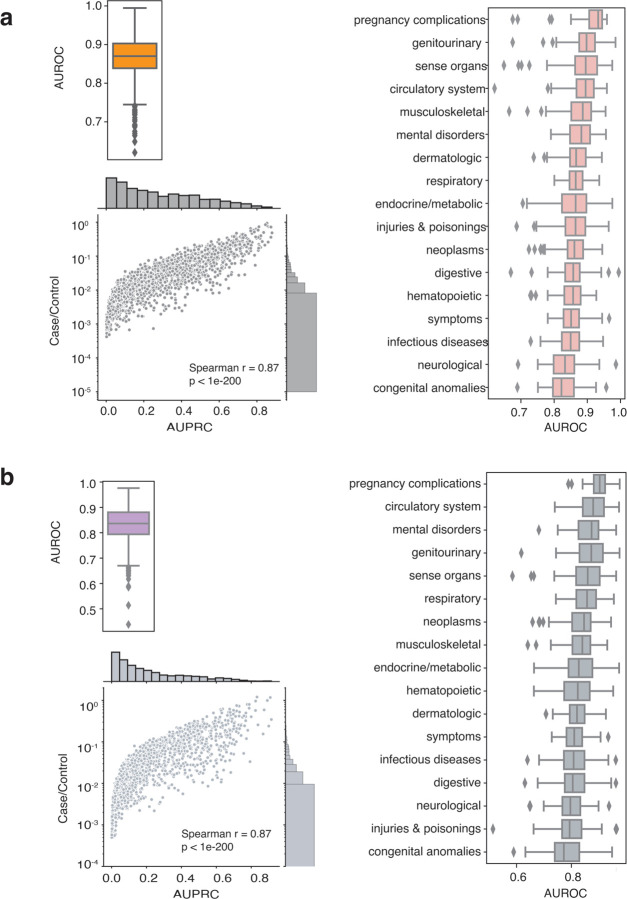
Model performance on (a) disease onset prediction and (b) bulk phenotyping Performances on disease onset prediction (a) and bulk phenotyping (b). Top right showing the boxplot of AUROC and bottom showing the relationship between sample size (case/control ratio) and AUPRC. On the right each boxplot represents AUROC distribution categorized by disease class according to phecodes.

Prediction of disease onset is crucial since it can help early diagnosis and provide risk measurement at a given time before the disease onset. Using the longitudinal embeddings of patient vectors, we first build simple logistic regression models to discriminate the disease versus the non-disease state (Figure S2a). We mapped ICD10 and ICD9 codes to phecodes as a standard reference of phenotypes ^28^. For a specific disease represented by phecodes, the patient vector after a diagnosis time point is denoted as cases (disease state), and the patient vectors before the diagnosis are controls (non-disease state). Across all 1855 unique phenotypes represented by phecodes, the simple logistic regression model achieved a median area under the receiver operating characteristic (AUROC) = 0.81, with the circulatory system group being the best and the pregnancy complications group the last (Figure S2b). The median area under the precision-recall curve (AURPC) is 0.80, which is not surprising, as the sample sizes of the cases and controls are approximately balanced (Figure S2b). This disease state versus non-disease state classification task can be seen as a baseline between “phenotyping” and “onset prediction” as it demonstrates the ability to differentiate between disease and non-disease states. However, it lacks timing in prediction. Therefore, we then performed disease onset prediction using patient vectors one year before disease onset (Figure 2a, Figure S3a). We observed a median AUROC of 0.87 for the disease onset task, with the best group being pregnancy complications (AUROC = 0.93) and the last group being congenital anomalies (AUROC = 0.82). These results surpassed the baseline disease status versus non-disease status prediction. We owe this exceeding performance to the fine-tuning tasks from the patient embedding model, where we trained the longitudinal vectors to predict the next event (Figure 1c, Figure S1). Though the sample size is heavily imbalanced within each phenotype for this task, we show that the AUPRC is highly associated with the case-to-control ratio (Figure 2a), indicating a stable performance when having enough samples. Together, these results suggest that the patient vectors can serve as features for disease forecasting tools or risk models while only using simple linear models.

Automated bulk phenotyping is ideal if no extra human effort is required, as developing high-performance phenotyping algorithms is usually iterative, time-consuming, and demands knowledge from domain expertise ^31,15^. Here, we explored the potential for using the longitudinal vectors as bulk-phenotyping tools and illustrated the process in Figure S3b, using phecodes as standard references. In detail, since each patient has multiple vectors corresponding to different time points, we took the arithmetic mean of those vectors as a compressed vector representation of each patient. This method resembles the chronological accumulation of all high-dimensional vectors and normalizes it by the number of steps (referred as summedup embedding later). The median AUROC of all 1855 phenotyping tasks is 0.84, a bit lower than the disease of onset task (Figure 2b). The best performance is for pregnancy complications (AUROC = 0.90) and the poorest for congenital abnormalities (AUROC = 0.77). Likewise, we observed a strong association between the cases-to-control ratio and the AUPRC (Figure 2b). Together, these two outstanding performances showed great potential for patient vectors to achieve excellent results in bulk phenotyping.

### Sub-phenotype identification using patient vectors clustering analysis

**Figure 3.**
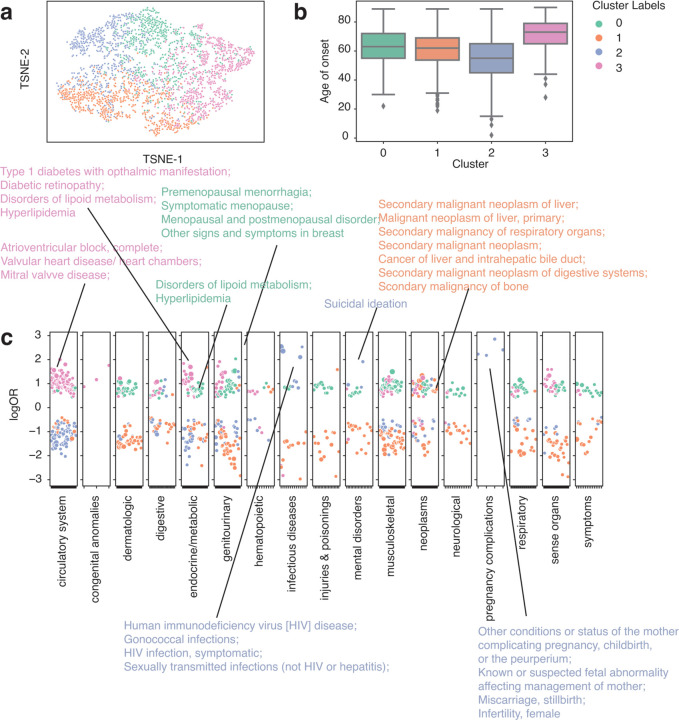
Clustering analysis identified subgroups with distinct comorbidity patterns in colorectal cancer patients (n = 2837) from the eMERGE cohort. a. TSNE plot of patient vectors colored by cluster groups defined using Gaussian Mixture Model (GMM) with optimal Bayesian Information Criteria (BIC). b. Box plot showing distribution of age of onset for individual CRC cluster groups. c. Comorbidity pattern enrichment plot grouped by disease classes (in x-axis) within each cluster group (represented by color). The y-axis indicates the log odds ratio of the comorbidity enrichment. Only statistically significant results are shown (p < 2e-5) after Bonferroni correction. Colored texts are used to highlight the top results within each cluster group.

Comorbidity, the simultaneous presence of two or more diseases or medical conditions, has a profound impact on an individual’s care plan, quality of life, and mortality ^32^. Using the summed-up embedding to represent individual patients, we show that within a well-defined single phenotype, the comorbidity status formed different clusters, exhibiting heterogeneity in comorbidity patterns (Figure 3). Using colorectal cancer (CRC) patients (identified using phecode 153) as an example (n = 2837), we identified 4 clusters according to the Bayesian Information Criterion (BIC) using Gaussian Mixture Models (GMM) (Figure S3). To characterize the comorbidity patterns within each cluster group, we fitted logistic regression models using one cluster group versus the rest strategy adjusted for age of onset, race, ethnicity, and sites (see Methods). Cluster 2 (median onset age = 51) has the earliest onset age and is strongly associated with HIV infection (phecode = 071) and a few pregnancy complications, representing a subgroup of female patients with immunodeficiency phenotypes. This association between HIV and CRC is not new and is more prevalent in women in a pooled result from 3 studies ^33–36^. Cluster 1 (median onset age = 62) is associated with secondary malignant neoplasm, which reflects cancer pleiotropy and late-stage cancer patients with metastasis ^37,38^. Cluster 0 (median onset age = 60) is enriched in genitourinary and endocrine diseases, including disorders of lipoid metabolism, menopause issues, and menorrhagia issues. Though endocrine and metabolic disease might be risk factors for CRC, and vice versa, a study has also shown a greater risk of CRC patients developing endocrine and metabolic diseases ^39,40^. Cluster 3 (median onset age = 72) group is the latest onset group, which has a strong pattern of the circulatory system and endocrine diseases, including atrioventricular block, valve heart disease, mitral valve diseases, diabetics, and hyperlipidemia. This cluster group aligns with existing findings that CRC patients have an increased risk of developing cardiovascular disease and heart failure ^41,42^.

Similarly, we performed GMM clustering on systemic lupus erythematosus (SLE) patients (n = 1806). We identified 4 clusters according to BIC and observed a wide range of disease patterns within these clusters (Figure S3). Cluster 0 has the lowest onset age (median onset age = 37) among all other groups and is associated with epilepsy. Cluster 1 has the highest median onset age (median onset age = 57) and is joint with skin cancer and eye diseases, such as glaucoma, cataracts, dermatochalasis, etc. Evidence shows that SLE patients can develop cataracts and many other eye diseases ^43,44^, and some might be due to medications ^44^. Cluster 2 has a median onset age similar to cluster 0 (median onset age = 40) and is enriched in pregnancy complications, including hemorrhage in early pregnancy, miscarriage, stillbirth, etc. Cluster 2 also has signs of infertility, irregular menstrual cycle, and developmental disorder (Figure 3). Though still unclear, numerous studies have tried to dissect the relationship between lupus and pregnancy complications and identified hormone-level abnormalities ^45,46^. Cluster 3 has a median onset age of 44 and is associated with renal diseases, including renal osteodystrophy, end-stage renal disease, chronic kidney diseases, etc. SLE is known to be a systemic disease, and cluster 3 reflects its systemic involvement in kidney disorder. When aggravated, it can lead to kidney failure. Using CRC and SLE as two case studies, we show that patient vectors can reveal distinct comorbidity patterns. Even within a single phenotype, there are diverse patterns of comorbidities. Thus, further evaluation and personalized care plan is required to improve healthcare.

Besides the above two phenotypes, we also present the visualization of other phenotypes that have a reasonable sample size. The interactive website is running on: https://ehrcluster.web.app/. Users can search for a phenotype of interest and visualize the subgroup clusters.

### External evaluation using UW EHR data identified similar comorbidity patterns and survival differences

To the best of our knowledge, most of the unsupervised patient representation learning models are only evaluated internally, without robust external validation. Here, we collected the EHR data at the University of Washington (UW) from 2000 to 2020, including n = 840,000 patients as external sources of validation to assess our model’s validity and robustness. First, albeit not comparable to the original model performances (Table 1), the transformer model trained on the eMERGE data still earned a reasonable performance in sequence recovery tasks running on UW patients, with a mean and median precision of 0.862 and 0.903, respectively. The mean and median recall are 0.852 and 0.896 (Table 3). Next, the performance of the sentence embedding model has an accuracy of 0.796±0.0023 in the is_same_patienttask and 0.742±0.0042 for the is_next_eventtask on the UW dataset. This result is interesting as it is comparable to the performance on the training set (eMERGE), and we can conclude that the performance is ideal for external validation (Table 4). Together, this evidence indicates that the transformer model can achieve great success in patient embedding internally and is also generalizable with the external UW cohort.

We then evaluated the performance of bulk phenotyping and disease onset prediction on the UW dataset (Figure S5). The median AUROC for the bulk phenotyping task and disease onset task are 0.83 and 0.84, respectively. We noticed a tiny performance drop in the UW datasets from comparing these two tasks to the original eMERGE dataset. Still, the performance is robust and surprisingly stable for an external evaluation.

**Figure 5.**
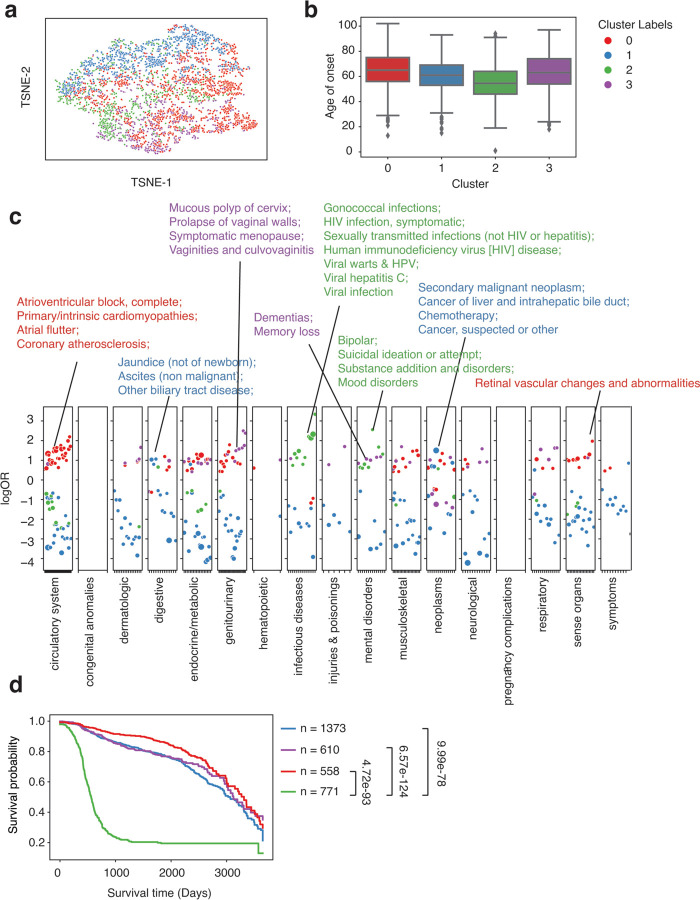
Clustering analysis identified subgroups with distinct comorbidity patterns in colorectal cancer patients from the UW cohort. a. TSNE plot of patient vectors colored by cluster groups defined using Gaussian Mixture Model (GMM) with optimal Bayesian Information Criteria (BIC). b. Box plot showing distribution of age of onset for individual CRC cluster groups. c. Comorbidity pattern enrichment plot grouped by disease classes (in x-axis) within each cluster group (represented by color). The y-axis indicates the log odds ratio of the comorbidity enrichment. Only statistically significant results are shown (p < 2e-5) after Bonferroni correction. Colored texts are used to highlight the top results within each cluster group. d. Kaplan-Meier curve showing 10-year overall survival differences across individual cluster groups.

Besides the model performance, bulk-phenotyping, and onset prediction, more importantly, we also performed the comorbidity analysis to see if we could re-identify similar disease patterns across distinct groups. We focused on SLE and CRC and compared the results between the UW cohort and the eMERGE cohort, aiming to reproduce the findings in the UW validation cohort. We discovered 4 clusters of CRC in the UW cohort (Figure 5A). One cluster (cluster 2, the median age of onset = 54.5) that has a relatively younger age of onset is enriched in infectious diseases (HIV infection, Viral hepatitis C, etc.) and a few mental disorders (Bipolar, Suicidal ideation or attempt, Mood disorder, etc.), identical to cluster 2 from the eMERGE cohort. (Figure 5b, c). Cluster 0 (median age of onset = 65) from the UW cohort is similar to cluster 3 from the eMERGE cohort (Figure 3), as both showed phenotype enrichment in circulatory systems (Atrioventricular block) and endocrine/metabolic diseases. Moreover, cluster 1 (median age of onset = 61) from the UW and eMERGE cohorts are both enriched in secondary malignant neoplasm, specifically, cancer of the liver and intrahepatic bile duct. Together, these results provided compelling evidence that our findings of disease subtypes from the training cohort (the eMERGE cohort) are stable and can be validated using the UW cohort externally. Again, analyzing the 10-year overall survival difference (Figure 5d), we found cluster 2 enriched in infectious diseases and mental disorders showed a significantly lower overall survival probability than the other 3 clusters. Searching through existing literature, though there are a few discussions about HIV and colorectal cancer risk, only one report used a meta-analysis method investigating the mortality rate of CRC patients with HIV with non-significant results, partially due to inadequate cases (n = 194) ^33^. One report also found HIV-infected cancer patients with elevated mortality rates ^47^. Our findings provide extra evidence supporting these research works and demonstrate the potential of uncovering new patterns in clinical outcomes among patients. Also, given that most research currently lacks the consideration of comorbidity patterns in outcome prediction and personalized medicine, our data shows that comorbidity patterns can provide crucial information in patient care and life expectancy.

**Figure 6.**
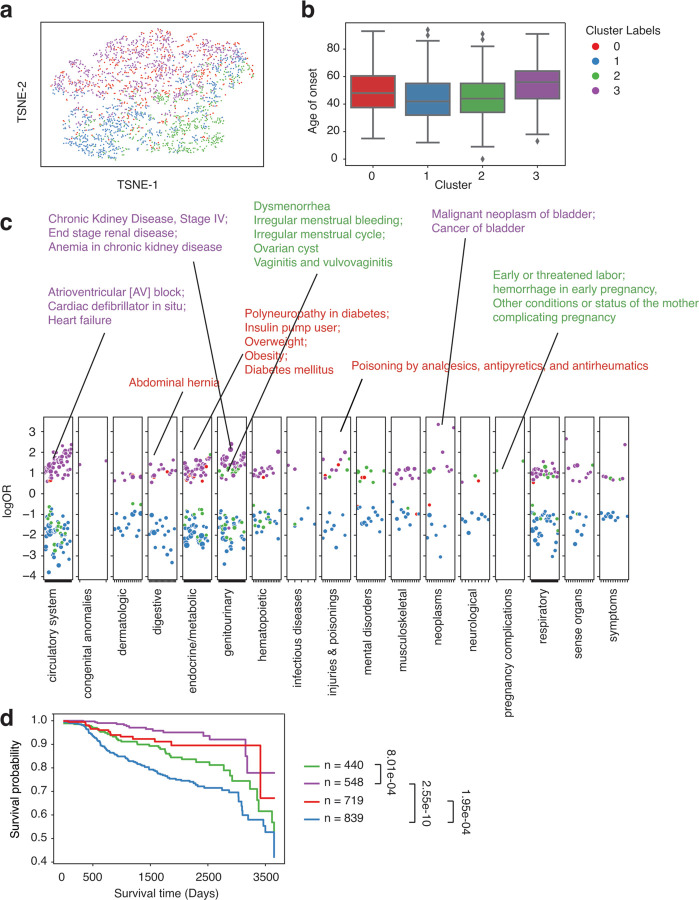
Clustering analysis identified subgroups with distinct comorbidity patterns in systemic lupus erythematosus patients from the UW cohort. a. TSNE plot of patient vectors colored by cluster groups defined using Gaussian Mixture Model (GMM) with optimal Bayesian Information Criteria (BIC). b. Box plot showing distribution of age of onset for individual SLE cluster groups. c. Comorbidity pattern enrichment plot grouped by disease classes (in x-axis) within each cluster group (represented by color). The y-axis indicates the log odds ratio of the comorbidity enrichment. Only statistically significant results are shown (p < 2e-5) after Bonferroni correction. Colored texts are used to highlight the top results within each cluster group. d. Kaplan-Meier curve showing 10-year overall survival differences across individual cluster groups.

In the UW cohort, we identified 4 clusters of SLE enriched for different comorbidities (Figure 6). Cluster 2 (median age of onset = 44) likewise, enriched with pregnancy complications, genitourinary, and a few mental disorders, has a relatively early onset age and is highly similar to what we identified in the eMERGE cohort. Cluster 0 (median age of onset = 48) is associated with endocrine/metabolic (such as diabetes, overweight, and obesity). Cluster 3 (median age of onset = 56) is associated with the circulatory system (Atrioventricular block, Cardiac defibrillator in situ, Heart failure, etc.), genitourinary (Chronic Kidney diseases, End stage renal disease, Anemia in chronic kidney disease, etc.), and a few neoplasms (Malignant neoplasm of bladder, Cancer of bladder). Cluster 1 (median age of onset = 42) does not have a unique pattern of enrichment of comorbidities. In short, we identified 4 clusters of SLE in the UW cohort, with three having distinct disease patterns. Two cluster groups have identical properties to what we have found in the eMERGE dataset, including pregnancy-complication-associated lupus (Cluster 2) and Renal-associated lupus (Cluster 3). Additionally, with available survival data in the UW cohort, we compared the 10-year overall survival among different cluster groups (Figure 6d). Among these comorbidity groups, cluster 1, which showed no comorbidity enrichment, has the lowest survival rate in 10 years. We reason that the low survival rate might partially be explained by a higher proportion of males in cluster 1 compared to other clusters (odds = 2.54, p = 1.42e-14). This is consistent with previous reports that male SLE patients suffer from a lower life expectancy compared to females ^48,49^. Then, we noticed that cluster 2 also showed a relatively lower survival rate than Cluster 3 (FDR = 8.01e-4). This result might imply that pregnancy-complication-associated lupus patients might need more follow-up and on-time treatment to improve their health outcomes and life expectancy. One national study also found that SLE women have 20-fold higher maternal death ^45^. Clusters 0 (Diabetes-associated SLE) and Cluster 3 (Renal-associated SLE) showed a better survival status, which also has a relatively late onset age, representing late-stage SLE when patients age since SLE usually involves multiple organ-level dysfunctions.

As cancers are the second leading cause of death with high levels of heterogeneity, we applied the GMM clustering framework to a wide range of cancer types and studied their survival differences (Figure S6).

### Distinct progression trajectory patterns among cluster groups

**Figure 7.**
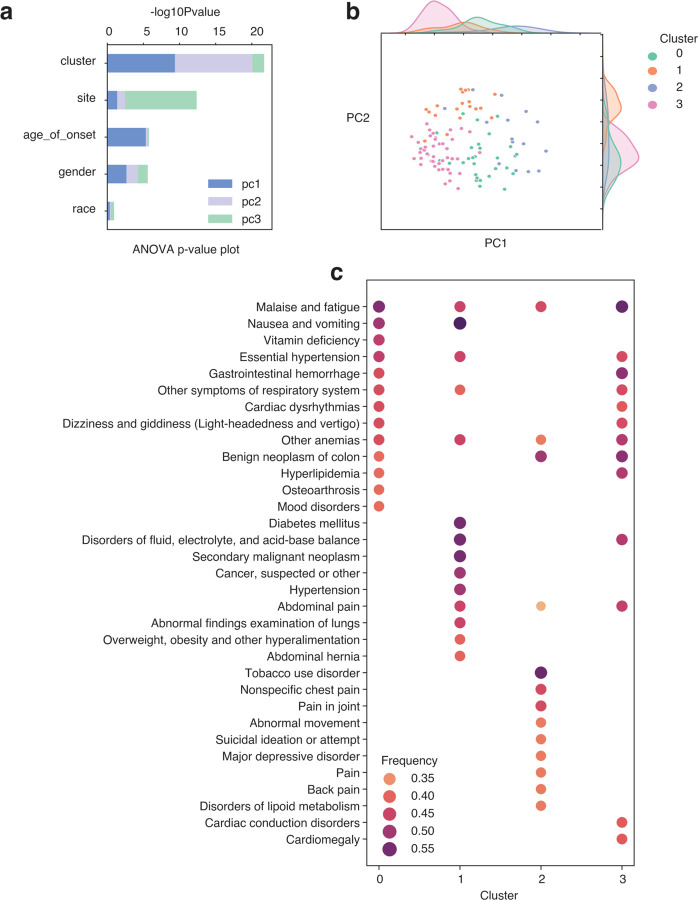
Longitudinal analysis revealed progression differences within each cluster group in CRC. a. Barplot showing ANOVA results in negative log10 P-value of each variable in y-axis explaining variances of PCs in different colors. b. Scatterplot showing the cluster groups in different colors are driven by PC1 and PC2. c. Dot plot indicating phenotype (comorbidity) gain (y-axis) within each cluster after CRC onset. Color scales are used to indicate the fraction within each cluster obtaining a new phenotype corresponding to the y-axis.

We further evaluated the potential of using longitudinal embedding vectors to study the progression of diseases. We used CRC (phecode = 153) as an example, using all available patients with 10-year longitudinal vectors after a diagnosis of CRC (n = 110). We first performed Principal Components Analysis (PCA) on the longitudinal vectors, trying to decompose the changes in disease progression and analyze the variance. We included the first three PCs, which composed 51% of the explained variances (Figure 7a). Using analysis of variance (ANOVA), including the age of onset, race, gender, site, and clusters as covariates, we found that PC1 and PC2 are explained majorly by the cluster groups we identified using GMM (see session), then the age of onset, gender, sites, and races (Figure 7a, b). And PC3 is explained mainly by sites (Figure 7b). This result suggests that the variation in disease progression longitudinally is also captured by the clusters, indicating that individual cluster groups also have a different disease progression track, meaning that the cluster groups we identified can project the disease trajectories. To understand the differences in progression, we then analyzed the emerging phenotypes following the onset of the disease, revealing substantial differences among cluster groups (refer to Figure 7C). Besides the consistent occurrences of “Malaise and fatigue” and “Other anemias” across all four clusters, a few phenotypes were also present in three out of four clusters. These included “Essential hypertension,” “Gastrointestinal hemorrhage,” “Other symptoms of the respiratory system,” “Benign neoplasm of the colon,” and “Abdominal pain.” The remaining emerging phenotypes displayed radical variations across all four clusters, indicating distinct progression trajectories and varied comorbidity patterns. Given the substantial disparities in the progression of the four clusters, we investigated their disease patterns before the onset of the CRC. Our investigation revealed their initial divergence, as illustrated in Figure S5. However, these disparities are not particularly prevalent; the maximum frequency across all four clusters is merely 21% (Figure S7). This frequency can subsequently escalate to as high as 55% after the onset of the disease (Figure 7). Together, these findings imply that disease progressions exhibit a high degree of heterogeneity, yet they still manifest discernible patterns in terms of comorbidities. This observation indicates varying disease risks and underscores the potential for personalized medicine approaches.

## Discussion

### Model architecture and performances

Mining information from the EHR has become a crucial topic for several meaningful downstream implementations, such as disease prediction, patient phenotyping, and personalized medicine ^14,50^. In this work, we developed a novel patient embedding method working with the EHR data. We integrated 3-step model architectures to achieve this complicated task and demonstrated a few downstream applications. We evaluated the model performance both internally and externally. Though the performance on the external UW EHR data dropped, the metrics score (Table 4) is still satisfying, experimentally demonstrating the robustness of our model. We did notice a drastic difference in the model performance on various lengths of patient vectors (Figure S8). This variation is related to the attention mechanism. The attention mechanism is designed for pairwise translation, where a sentence usually consists of a proper number of words. Therefore, when a patient sequence sparsely contains very few codes, the model lacks a strong co-occurrence pattern to recover the original codes.

Several other studies have explored the potential of representation learning to characterize diseases using the EHR with various machine learning architectures ^10,11,13,51,52^. Most of these studies use different metrics and disease annotations, making it challenging to compare the performances. However, we did notice a trend of more complicated model architectures and downstream applications within the development of this new area, indicating a promising future for EHR representation learning. Among them, our model used a novel vocabulary embedding strategy to represent the diagnosis and procedure codes in the onset frequency domain. Our model showed more effortless components of embedding (only diagnosis and procedure codes and high computational efficiency) but still achieved vigorous performances. To our knowledge, we are the first group using a complex source of EHR data across 12 sites to perform the representation learning, leading to a thorough and more generalized patient representation model. Additionally, we seem to be the only group that examined the model performance externally, validating the results and demonstrating the reproducibility using a local UW EHR dataset.

### Implications and clinical importance

We demonstrated that a simple linear combination of the embedded features can manage disease onset predictions and bulk phenotyping tasks. The disease onset prediction itself is not only an evaluation of the embedding but also shedding new light on an automatic prediction tool for the alert system in the EHR regarding patient risks of future diseases.

In disease comorbidity analysis, we applied cluster algorithms and detected distinct comorbidity patterns within CRC and SLE. Meanwhile, we reproduced similar cluster results and revealed distinctions in overall survival in the UW cohort. In the progression analysis, we show that each cluster is associated with distinct phenotype gain, which suggests that the cluster groups are progressively different, meaning that we are not capturing a static moment but a continuous variation. Moreover, this unsupervised method is data-driven and thus not limited to existing knowledge for EHR-based risk prediction and can uncover new disease patterns and potentially explain the progression differences of highly heterogeneous diseases. Further analysis with UW EHR data identified overall mortality disparities among those disease cluster groups, showing room for further investigation of health outcome variations.

Together, these results suggest that partitioning patients into different subgroups can assist in identifying discrepancies in disease progression and critical health outcomes using EHR data. Though more efforts are needed to understand the complex comorbidity relationship, implementing an intelligent clinical decision support system to facilitate personalized medicine is feasible, leveraging the abundant patient data within the EHR. For instance, when the system notices that an HIV patient has recently been diagnosed with CRC, which is an extremely high risk, an early warning can inform the severity of the co-occurrence of these two phenotypes to facilitate on-time treatment. Thus, this system can identify urgency and achieve early healthcare to increase life expectancy. Besides actions in clinical application, identifying comorbidity patterns within a phenotype might indicate pathological, etiological, behavioral, or environmental similarities between co-occurring phenotypes. These comorbidity patterns can facilitate fundamental scientific advances in identifying molecular signatures of diseases and thus help us understand the mechanism better.

### Limitations, Strengths, and Future Directions

Our model has a few notable limitations. First, our model only included diagnosis and procedure codes as embedding building blocks, lacking medications, lab values, observations, and clinical notes due to the limitation of data sources. Without these variables, our model might lose certain meaningful information and limit the downstream analysis on medications, labs, etc. Besides, we used phecodes as surrogate phenotypes. Though phecodes have demonstrated their efficiency in large-scale EHR-based genetic studies, they might lack granularity and not be appropriate for some complicated phenotypes, such as depression^53^. Finally, our patient data drawn from the eMERGE consortium might contain potential ascertainment bias during patient recruitment, meaning that there might be population structures that can’t represent the general population of the United States. However, on the other hand, with only diagnosis and procedure codes available, our model still demonstrated great performances in several downstream analyses, such as bulk phenotyping, disease forecasting, comorbidity pattern study, and progression analysis. This is not surprising, as diagnoses and procedures are the most crucial information within the EHR for many downstream tasks. Though phecodes are new and still in development, there is evidence that phecodes can reproduce genetic findings and serve as a great proxy for phenotypes. To adjust the potential biases caused by individual sites, we always included sites as covariates in our statistical analysis. Most importantly, we demonstrated the external validity of our model using the UW dataset and exhibited the robustness of performances with experimentally reproduced stable disease patterns invariant of cohorts.

In summary, our study created a novel model architecture for patients representing learning using the EHR data, demonstrating the power of bulk phenotyping, disease forecasting, and comorbidity variations. More importantly, we showed that the disease trajectory is inherently different within each cluster, providing insights for studying the progression of the disease to understand its etiology and pathology. Future works may consider two directions among the others. The first is to consider integrating labs, medications, and fundamental observations such as vital signs into the current model to boost the performance and perform complicated downstream analysis toward health outcomes. The second is to perform detailed subgroup characterizations, including implementing topic modeling on patients and complex clinical outcome analysis^25,54,55^.

## Supplementary Material

Supplement 1

## Figures and Tables

**Figure 4.**
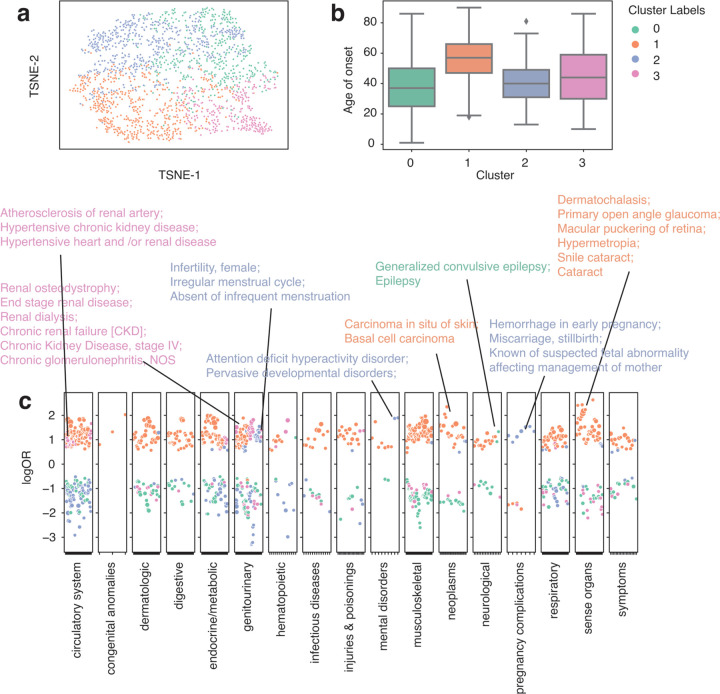
Clustering analysis identified subgroups with distinct comorbidity patterns in Systemic lupus erythematosus patients (n = 1806) from the eMERGE cohort. a. TSNE plot of patient vectors colored by cluster groups defined using Gaussian Mixture Model (GMM) with optimal Bayesian Information Criteria (BIC). b. Box plot showing distribution of age of onset for individual SLE cluster groups. c. Comorbidity pattern enrichment plot grouped by disease classes (in x-axis) within each cluster group (represented by color). The y-axis indicates the log odds ratio of the comorbidity enrichment. Only statistically significant results are shown (p < 2e-5) after Bonferroni correction. Colored texts are used to highlight the top results within each cluster group.

**Table 1.** Evaluation of sequence recovery performance

	median	mean
precision	0.958	0.917
recall	0.937	0.895

**Table 2.** Evaluation of the is_same_patient and the is_next_event performance for the sentence-embedding model

	is_same_patient	is_next_event
Accuracy	0.797±0.0016	0.769±0.011

**Table 3.** External validation of the is_same_patient and the is_next_event performance for the sentence-embedding model using the UW cohort

	median	mean
precision	0.903	0.862
recall	0.896	0.852

**Table 4.** Evaluation of the is_same_patient and the is_next_event performance for the sentence-embedding model using the UW cohort

	is_same_patient	is_next_event
Accuracy	0.796±0.0023	0.742±0.0042

## Data Availability

The data used for this study are available from request at the eMERGE Network.
