# Supplementary material for: Language-model-based patient embedding using electronic health records facilitates phenotyping, disease forecasting, and progression analysis
Source: Res Sq. 2024 Sep 23:rs.3.rs-4708839. Preprint. [Version 1] doi: 10.21203/rs.3.rs-4708839/v1 (PMC11469380; doi:10.21203/rs.3.rs-4708839/v1)

Supplementary Figure

Supplementary Figure 1. Illustration of the model architecture

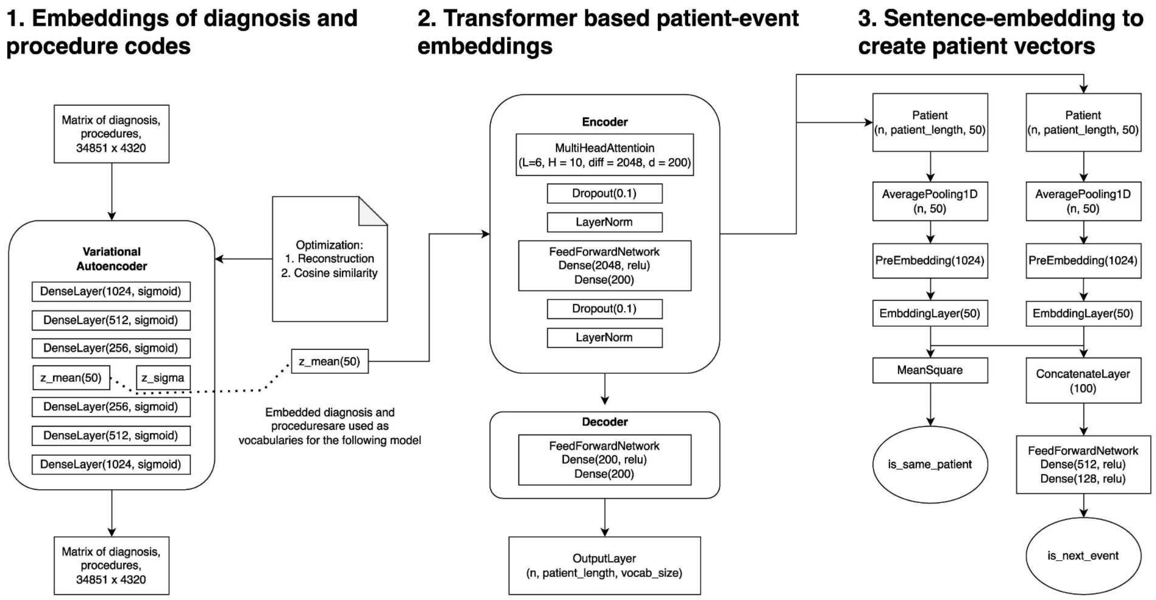

Supplementary Figure 2. (a) Disease onset prediction illustration and (b) disease versus non-disease state prediction.

a. Illustration of predicting disease versus non-disease status using patient vectors
b. Performances of disease versus non-diseases state prediction. Boxplot on the left side showing accuracy (ACC), AUROC and AUPRC distribution for all 1855 phenotypes. Boxplot on the right showing AUROC and AUPRC grouped by different disease classes.

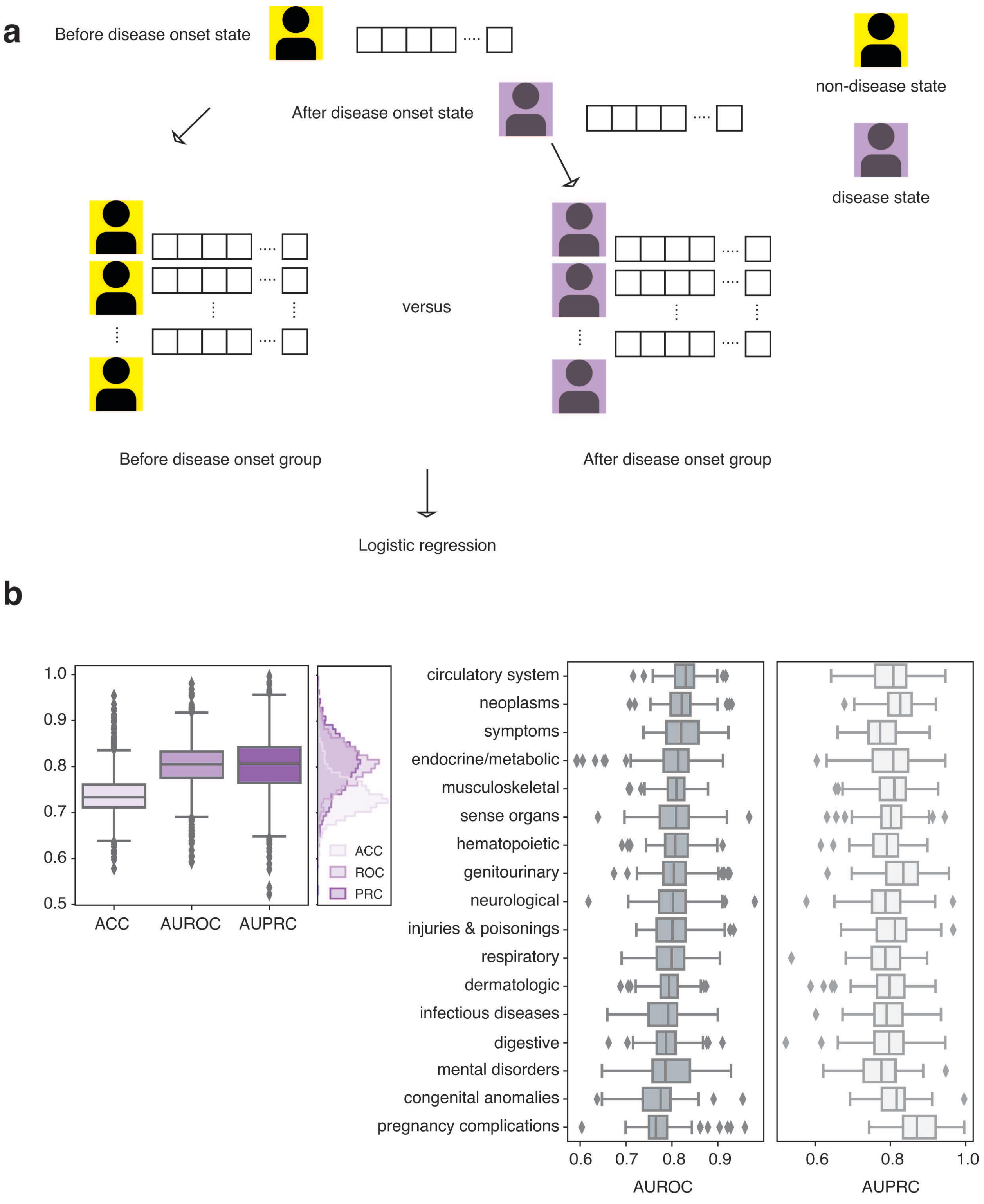
a
Before disease onset state
After disease onset state
non-disease state
disease state
versus
Before disease onset group
After disease onset group
Logistic regression
b
1.0
0.9
0.8
0.7
0.6
0.5
ACC
AUROC
AUPRC
ACC
ROC
PRC
circulatory system
neoplasms
symptoms
endocrine/metabolic
musculoskeletal
sense organs
hematopoietic
genitourinary
neurological
injuries & poisonings
respiratory
dermatologic
infectious diseases
digestive
mental disorders
congenital anomalies
pregnancy complications
0.6 0.7 0.8 0.9
AUROC
0.6 0.8 1.0
AUPRC

Supplementary Figure 3. Illustration of onset prediction and bulk phenotyping

a. Illustration each step of onset prediction tasks

b. Illustration each step of bulk phenotyping tasks

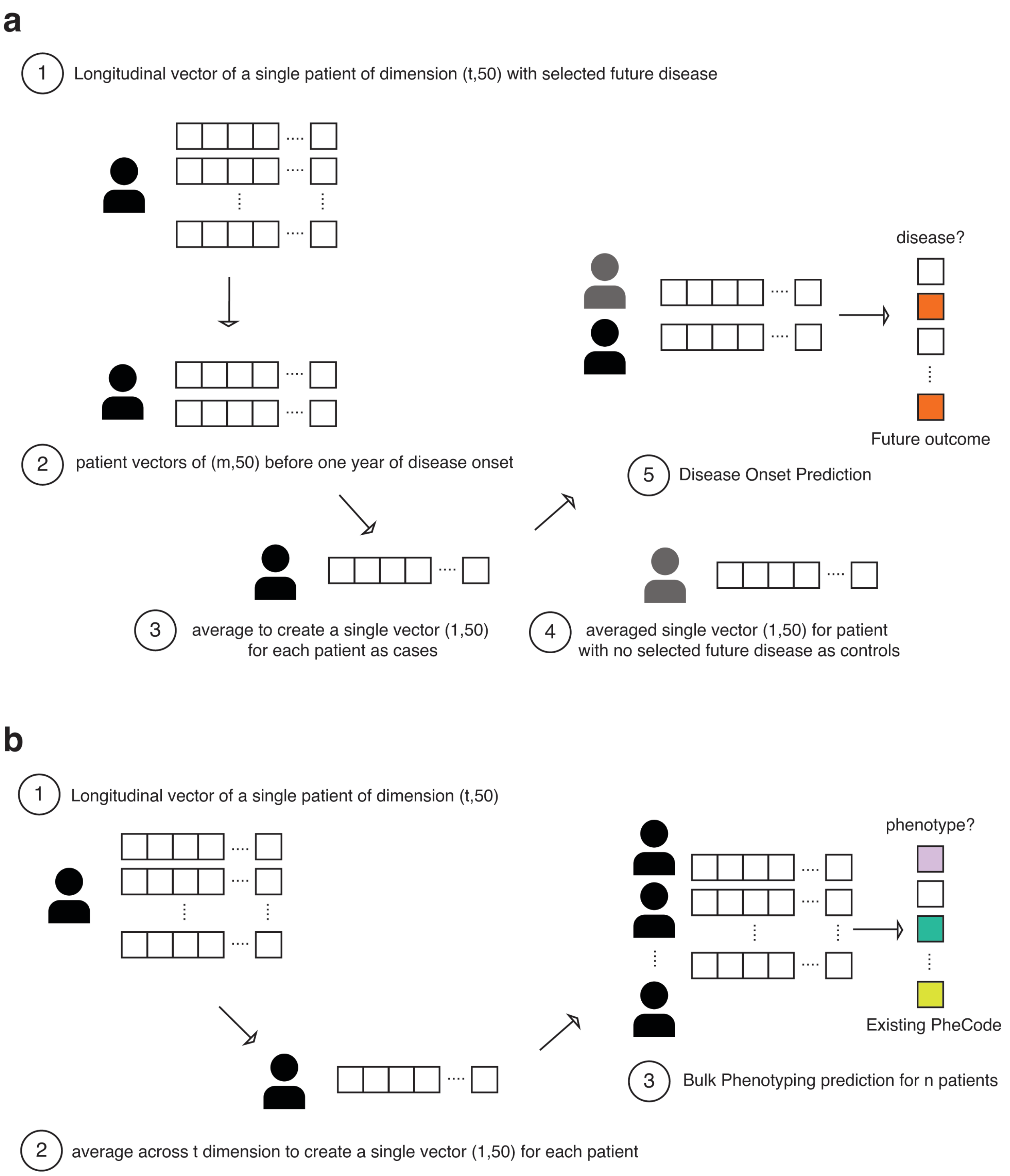

Supplementary Figure 4. Bayesian Information Criteria (BIC) curve for model selection. (a, b) for eMERGE cohort and (c, d) for the UW cohort

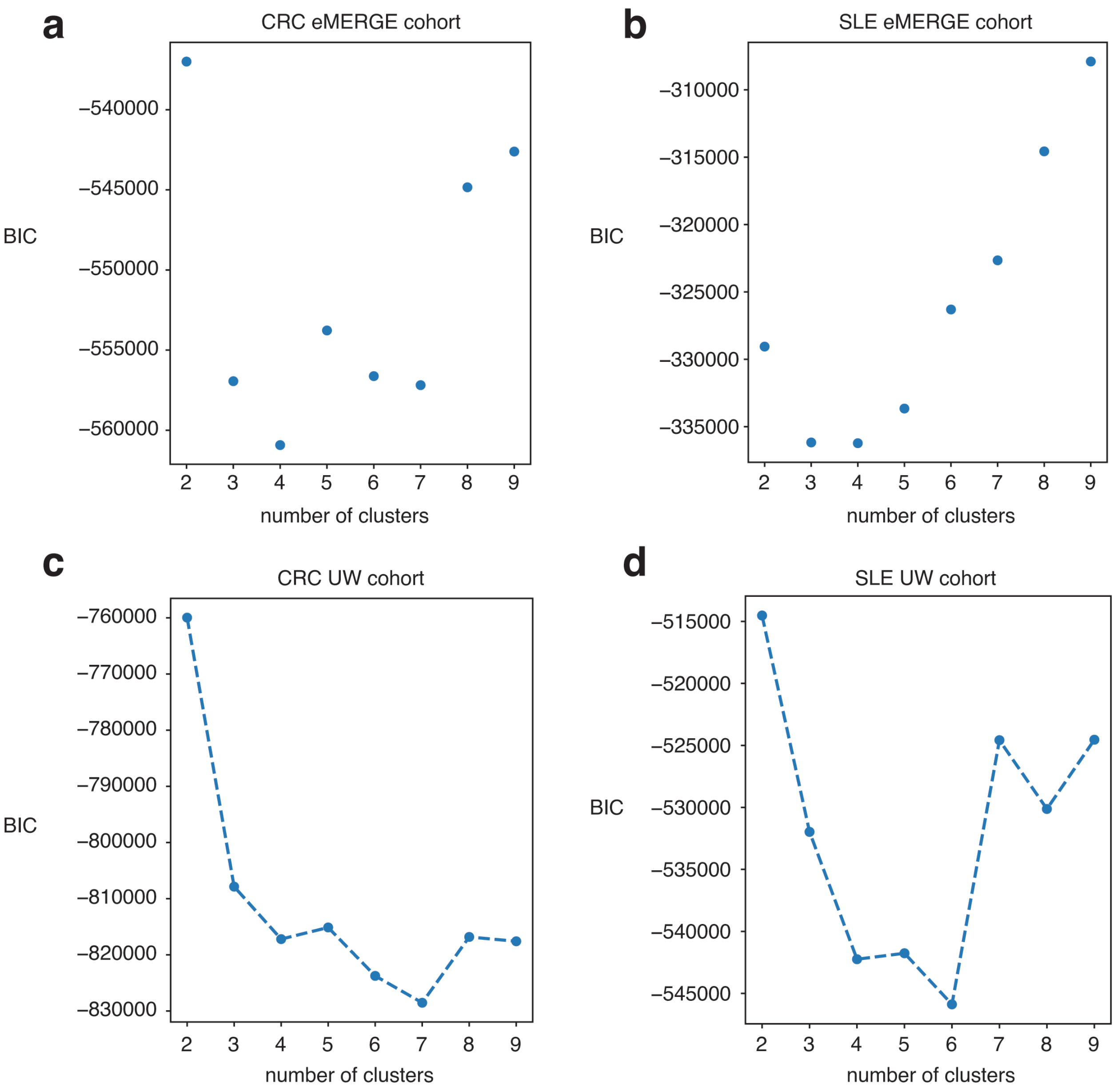

Supplementary Figure 5. Model performance on (a, b) disease onset prediction and (c, d) bulk phenotyping using the external UW cohort.

Performances on (a, b) disease onset prediction and (c, d) bulk phenotyping. Left side of each panel (a, c) shows the box plot representing AUROC distribution categorized by disease class according to phecodes and the right side (b, d) shows the relationship between sample size (case/control ratio) and AUPRC.

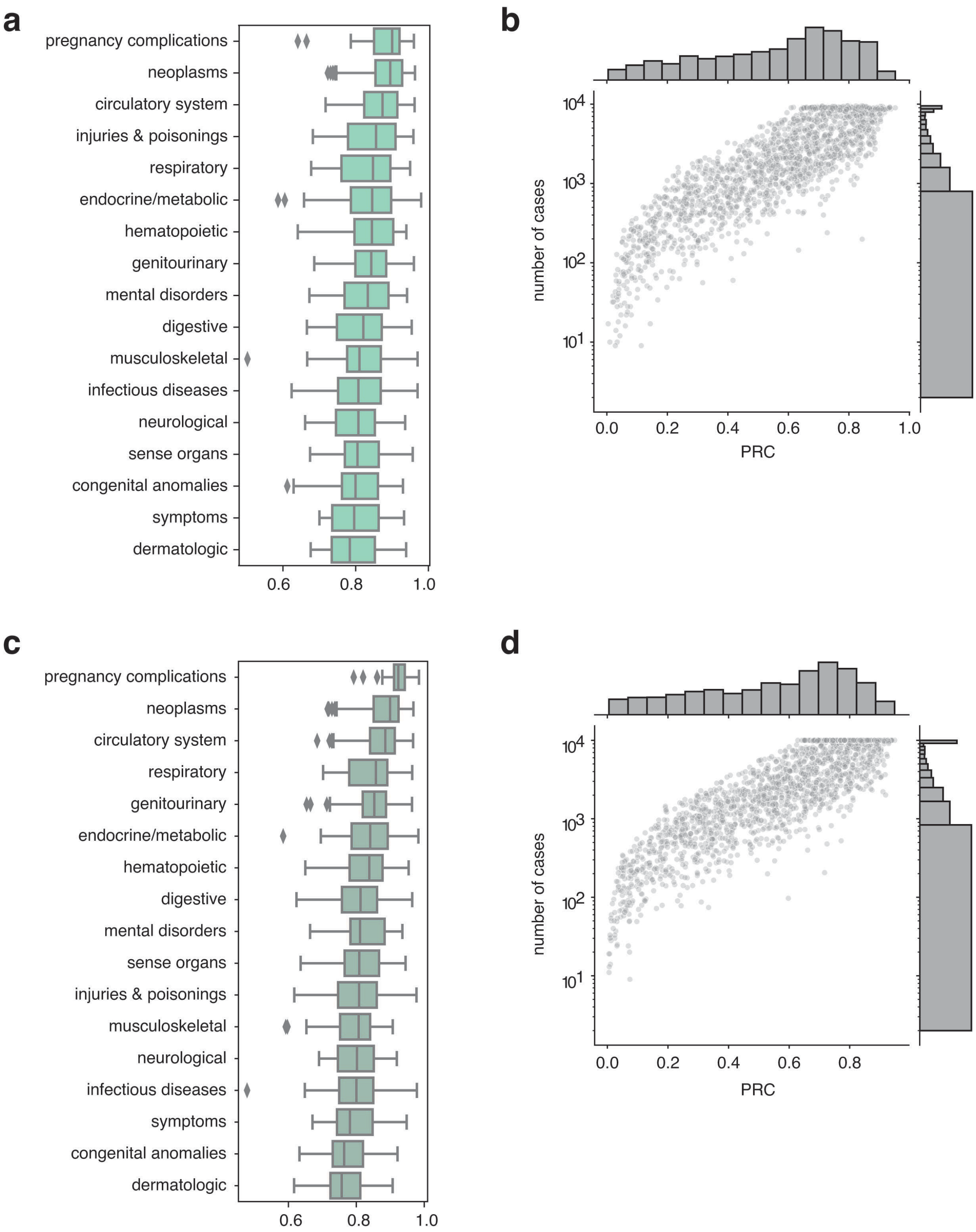
a
pregnancy complications
neoplasms
circulatory system
injuries & poisonings
respiratory
endocrine/metabolic
hematopoietic
genitourinary
mental disorders
digestive
musculoskeletal
infectious diseases
neurological
sense organs
congenital anomalies
symptoms
dermatologic
0.6
0.8
1.0
b
number of cases
10^4
10^3
10^2
10^1
0.0
0.2
0.4
0.6
0.8
1.0
PRC
c
pregnancy complications
neoplasms
circulatory system
respiratory
genitourinary
endocrine/metabolic
hematopoietic
digestive
mental disorders
sense organs
injuries & poisonings
musculoskeletal
neurological
infectious diseases
symptoms
congenital anomalies
dermatologic
0.6
0.8
1.0
d
number of cases
10^4
10^3
10^2
10^1
0.0
0.2
0.4
0.6
0.8
PRC

Supplementary Figure 6. Survival curve of multiple cancer types in the UW cohorts based on suggested clustering results revealing drastic differences in survival times.

Kaplan-Meier curves reveal differences of 10-year overall survival within each cluster group of multiple cancer types defined by phecodes.

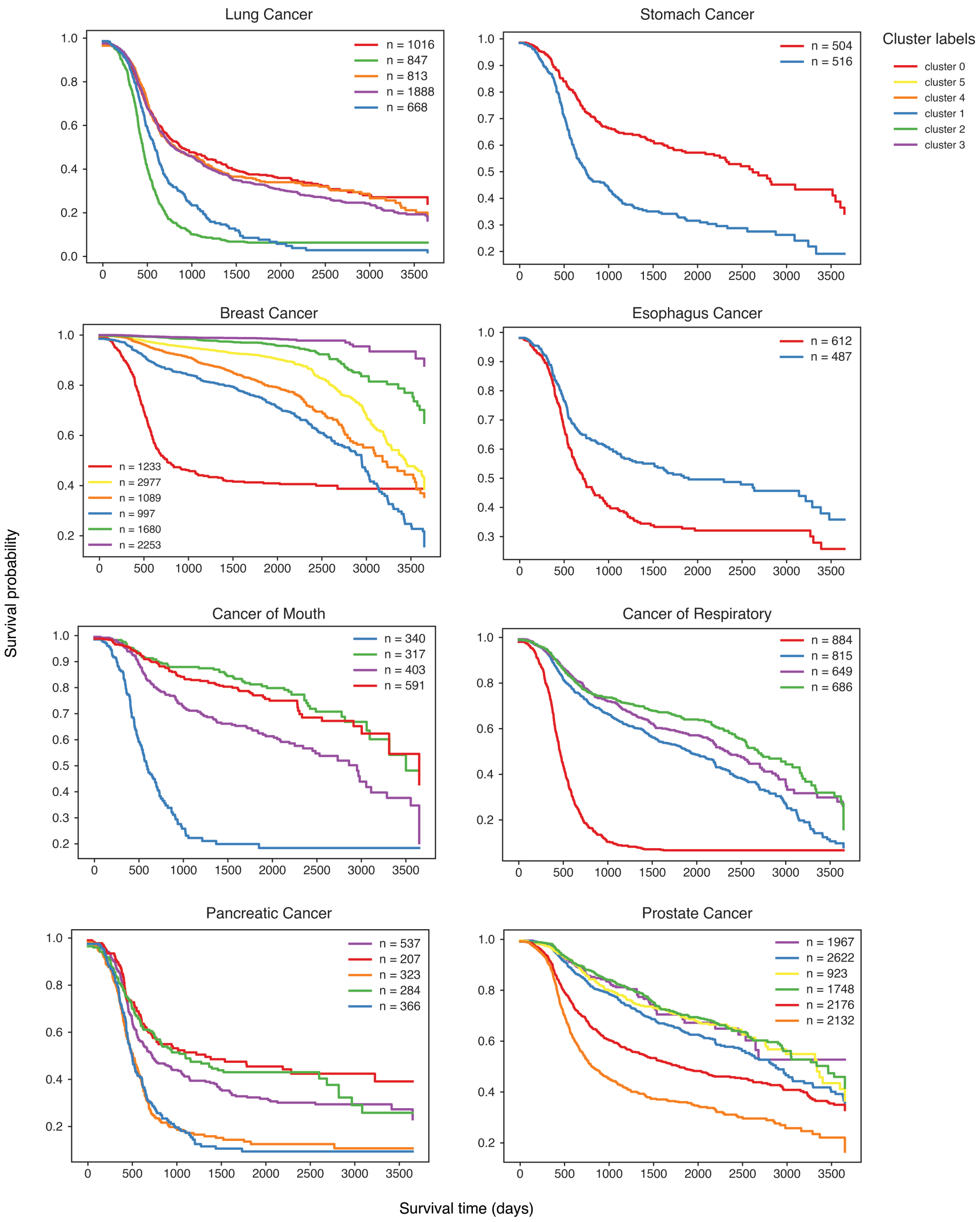
Lung Cancer
Stomach Cancer
Cluster labels
cluster 0
cluster 5
cluster 4
cluster 1
cluster 2
cluster 3
n = 1016
n = 847
n = 813
n = 1888
n = 668
n = 504
n = 516
Breast Cancer
Esophagus Cancer
n = 1233
n = 2977
n = 1089
n = 997
n = 1680
n = 2253
n = 612
n = 487
Cancer of Mouth
Cancer of Respiratory
n = 340
n = 317
n = 403
n = 591
n = 884
n = 815
n = 649
n = 686
Pancreatic Cancer
Prostate Cancer
n = 537
n = 207
n = 323
n = 284
n = 366
n = 1967
n = 2622
n = 923
n = 1748
n = 2176
n = 2132
Survival probability
Survival time (days)

Supplementary Figure 7. Comorbidity differences within each cluster group in CRC before onset of CRC. Dot plot indicating phenotype (comorbidity, y-axis) within each cluster before CRC onset. Color scales are used to indicate the fraction within each cluster that has a phenotype corresponding to the y-axis.

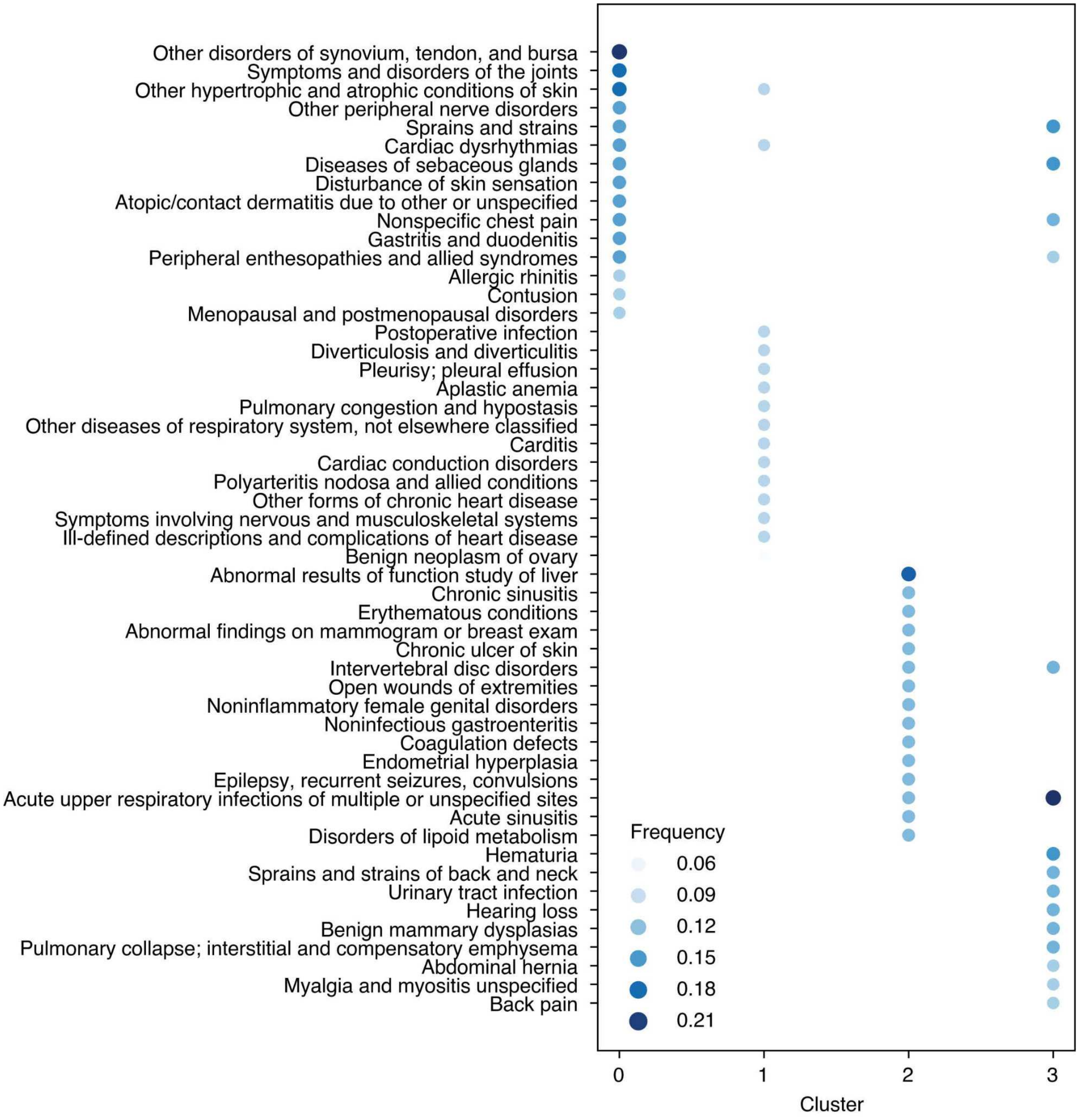

Supplementary Figure 8. Performance (precision and recall) differences in patient events with different numbers of codes.

a - b. Precision (a) and recall (b) based on the numbers of codes binned into ranges in each patient event (x-axis).

c - d. Same relational plot of a - b but in continuous manner without binning numbers of codes into ranges.

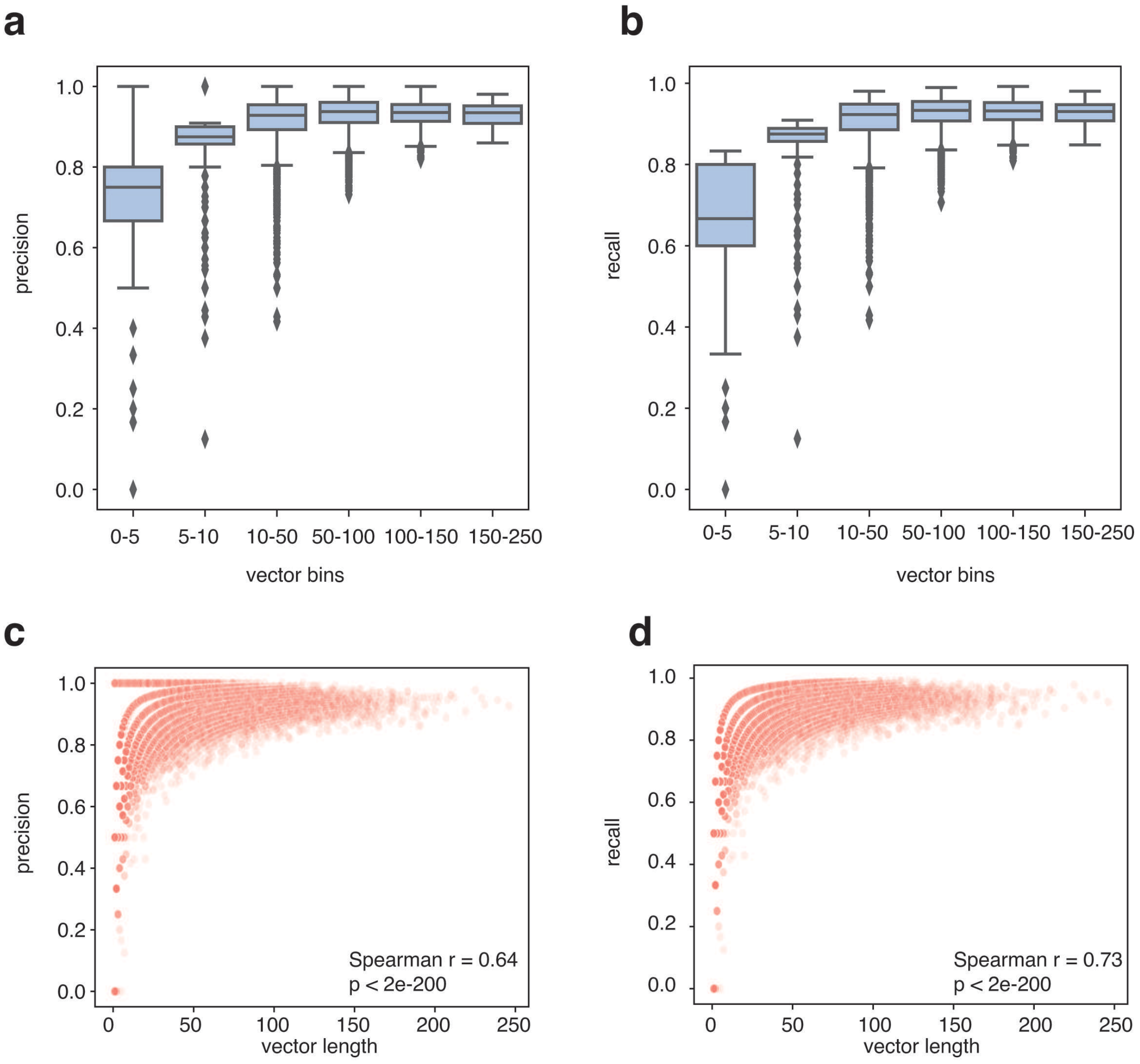

Supplement: Supplement 1 [file NIHPPRS4708839V1-supplement-1.pdf]
